# Breast cancer screening with mammography in women aged 40–49 years: Impact of length of screening interval on effectiveness of the program

**DOI:** 10.1177/0969141320918283

**Published:** 2020-05-13

**Authors:** Zheng Mao, Lennarth Nyström, Håkan Jonsson

**Affiliations:** 1 Department of Radiation Sciences, Oncology, Umeå University, Umeå, Sweden; 2 Department of Public Health and Clinical Medicine, Epidemiology and Global Health, Umeå University, Umeå, Sweden

**Keywords:** Breast cancer, mammography, mortality, evaluation, screening interval

## Abstract

**Objectives:**

To estimate the impact on the effectiveness of Swedish breast cancer screening program in women aged 40–49 years of shortening the screening interval from 21 months to 18 or 12 months.

**Methods:**

The reduction in breast cancer mortality among participants in screening with mammography was previously estimated in the Swedish SCReening of Young women (SCRY) study to be 29%. The expected increased effectiveness with a hypothetical shorter screening interval than the average of 21 months in SCRY was calculated using data about the women who died from breast cancer even though they participated in the SCRY program.

**Results:**

During the study period, 547 women who participated in the index screening round died from breast cancer. Shortening the screening interval to 18 months led to an improved effectiveness of 0.7–3.9% considering interval cancers only and of 1.3–7.6% considering screening-detected cancers only, and for both interval and screening-detected cancers the improvement was 1.9–11.5% when the assumed mortality reduction for the deceased cases varied from 5% to 30%. Shortening the screening interval to 12 months increased the effectiveness by 1.6–9.8% for interval cancers and by 2.9–17.4% for both interval and screening-detected cancers.

**Conclusion:**

Shortening the screening interval for women aged 40–49 years to 18 or 12 months might further reduce the breast cancer mortality rate.

## Introduction

The European Commission recommends biennial mammography screening for women aged 50–69 years (EU Guidelines). Most countries in Europe follow the recommendation, but some countries also invite women younger than 50 years because both randomized controlled trials (RCTs) and observational studies have indicated that inviting these women might reduce breast cancer mortality. RCTs in the US (HIP),^1^ Scotland,^2^ Canada (CNBSS I),^3^ the UK (Age Trial),^4^ and Sweden^5^ all included women 50 years and younger, but the trials were too small to draw any conclusions about the efficacy of screening. However, only CNBSS I^3^ and the Age Trial^4^ had the aim to study this group specifically, but neither of them showed a significant effect. Overviews and meta-analyses of RCTs with women aged 40–49 at randomization have also been published. Nelson et al.^6^ reported a 15% breast cancer mortality reduction based on eight trials (the Age Trial not included) (RR = 0.85; 95% CI: 0.75–0.96). The screening interval in the trials varied from 12 to 28 months.

The Swedish mammography service screening program was initiated in 1986, and the effectiveness of mammography screening for women aged 40–49 years was estimated in a nationwide study using the SCReening of Young women (SCRY) database. This was similar to a natural experiment in that in half of the country the lower age limit of invitation was 40 years, while in the other half it was 50 years. With a 16-year follow-up and 21-month screening interval on average, there was a significant reduction in breast cancer mortality among the invited women (RR = 0.74; 95% CI: 0.66–0.83) and among those attending mammography screening (RR = 0.71; 95% CI: 0.62–0.80) based on 7.3 and 8.8 million person-years in the intervention and control group, respectively.^7^

Because RCTs and observational studies of inviting woman aged 40–49 years to mammography screening have resulted in both significant and non-significant results, there has been a discussion regarding the optimal screening interval. The main argument for a shorter screening interval (less than two years) is that fast growing tumors and dense breasts are more common in younger women.^8,9^

Two randomized trials have been performed comparing the length of the screening interval. In the Co-ordinating Committee on Cancer Research trial in the UK, 99,389 women aged 50–62 years who had been invited to a prevalent screen were randomly allocated after the scheduled prevalent screen to the study arm (annual screens) or to the control arm (invited to a single screen three years later).^10^ In total, 37,530 women in the study arm and 38,492 in the control arm attended the prevalent screen. The tumor size was significantly smaller in the study arm, but there was no statistically significant difference in nodal status, grade, or predicted 10–15-year survival.

In Turku, Finland, women aged 40–74 years of age were invited to biennial screening except for the age group 40–49 years, which was randomized to either one-year or three-year intervals.^11^ During the study period 1987–1994, out of 59 invasive cancers diagnosed in the annual screening arm, 16 (27%) were interval cancers, while out of 44 invasive cancers diagnosed in the triennial screening arm, 17 (49%) were interval cancers. Thus, there was no difference in the number of interval cancers, but the proportion of screen-detected cases was higher with the one-year interval.

In the Screening Mammography Programme of British Columbia (SMPBC), the screening interval was changed from one year to two years for women aged 50–79 years.^12^ There were 152,226 women screened biennially and 184,764 screened annually. The number of cases of positive lymph nodes and ductal carcinoma in situ increased (RR = 1.2, 95% CI: 1.1–1.4 and RR = 1.3, 95% CI, 1.1–1.5, respectively), while no significant differences were found regarding interval cancer, screen-detected cancer, tumor size ≥20 mm, grade, or breast cancer mortality.

Several observational studies have compared screening performance parameters between women with individually varying intervals. Hunt et al. in the US followed 24,211 women aged 40–79 years (in which 40% were younger than 50 years), and they showed that annual screening resulted in a lower recall rate (*p* < 0.0001) and detection of smaller tumors (*p* = 0.04) than biennial screening.^13^ Data from the US Breast Cancer Surveillance Consortium showed that there was a significantly higher risk of late-stage cancer with positive lymph nodes with biennial vs. annual screening of women aged 40–49 years (OR = 1.4, 95% CI: 1.0–1.8).^14^ Evaluation of a breast cancer screening program (for women aged 50–70 years) was performed in a study in England, Wales, and Northern Ireland, in which the interval cancer rates at 0–12 months, 13–24 months, and 25–36 months after a negative screen were 0.55, 1.13, and 1.22 per 1000 women screened, respectively.^15^

In summary, evidence is lacking on the impact of the length of the screening interval on breast cancer mortality in women aged 40–49 years. The aim of this study was to estimate the impact of shortening the screening interval to 18 or 12 months by using data and results from the SCRY study of women aged 40–49 years in Sweden with an average screening interval of 21 months.

## Methods

The SCRY study collected information on the initiation and organization of the service-screening program with mammography (SSPM) in all counties in Sweden by sending a questionnaire to the screening centers. The reported screening intervals varied between counties from 18 to 26 months, and the average was 21 months. The average follow-up was 16 years.

All women diagnosed with breast cancer in the age group 40–49 years after the start of the SSPM were retrieved from the Cancer Register and linked to the Cause of Death Register to get information on the date and cause of death. For women who died from breast cancer, data on the date of invitation and attendance in the index round, defined as the last invitation to screening before the diagnosis, were collected from the screening centers. Incidence-based breast cancer mortality rates in the study group cohorts of women invited to screening were compared with corresponding rates in the control cohorts of women not invited to screening.

**Figure 1.**
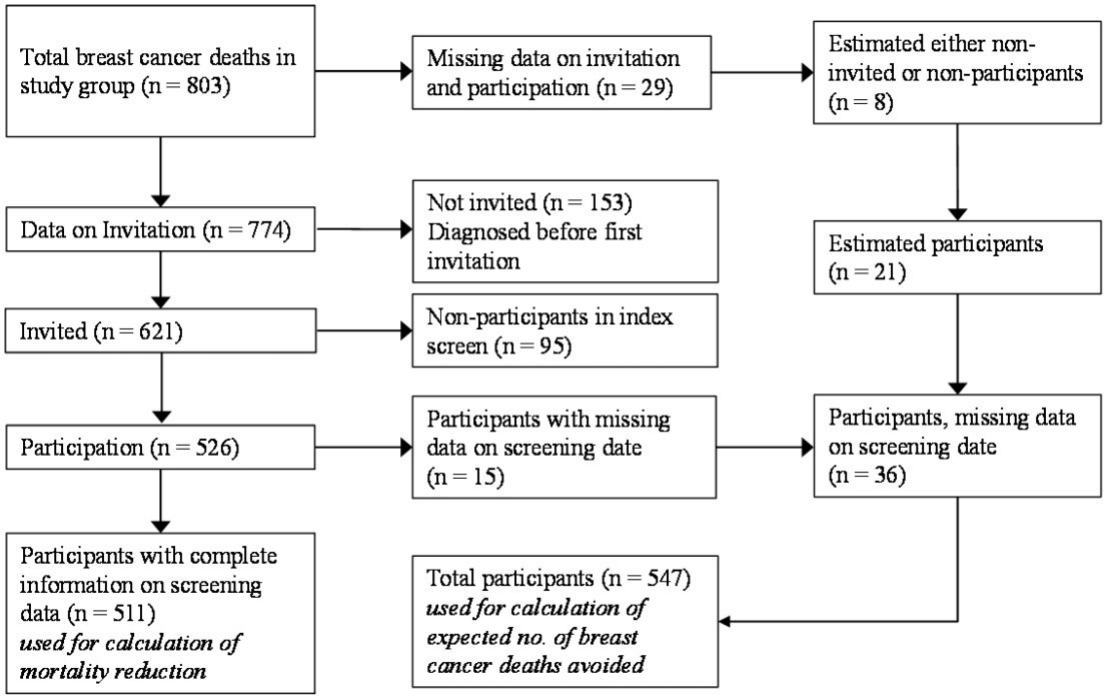
Flowchart of the number of breast cancer deaths included in the study.

In the current study, we utilized the fact that the effectiveness of participating in screening vs. not being invited was estimated at 29% (RR = 0.71, 95% CI: 0.62–0.80).^7^ Only the study groups, i.e. cohorts from areas inviting women 40–49 years to screening, were used. In total, 803 women died with breast cancer as the underlying cause of death during the follow-up. Information on invitation to and participation in screening was missing for 29 deaths. These were all from the same hospital where no records were available, and the follow-up time for that area was 20 years. Out of the 774 remaining women, 153 were diagnosed with breast cancer before their first invitation to screening because all cases were followed from the start of the screening programs. Furthermore, 95 did not participate and 15 of the participating women lacked an explicit screening date for the index round. Thus, 511 were participants with complete data. For the 29 with missing data on invitation and participation, we estimated the probability of being invited during 20 years of follow-up instead of 16 years at 0.835 (621 × 1.25/(153 + 621 × 1.25)) and assumed the participation rate was 84.7% (526/621). This resulted in 21 (0.835 × 0.847 × 29) estimated participants. Thus, an additional number of 36 participants (21 + 15) could be added to give a total of 547 (Figure 1).

We assumed that some of the women who participated in the index round and died from breast cancer in the SCRY study might have benefitted from a shorter screening interval with earlier cancer detection. By identifying these cases, the improved screening effectiveness could be calculated given a certain mortality reduction.

**Figure 2.**
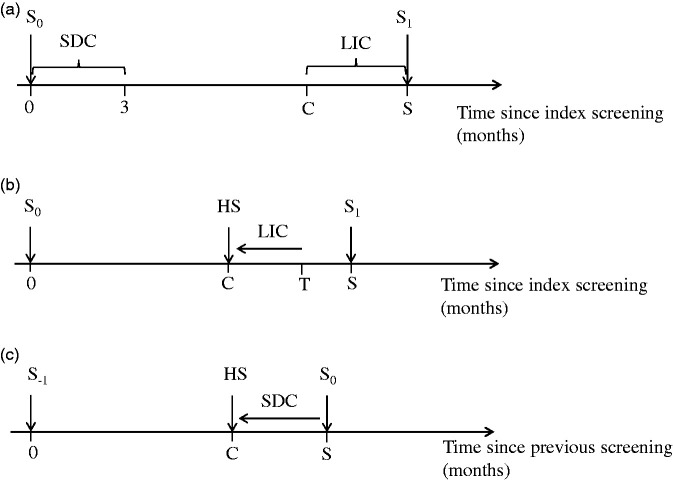
Illustration of the definition of screen-detected cancer (SDC) and late interval cancer (LIC) and possible earlier detection of LIC and SDC with a shorter screening interval. (a) The index screening S_0_ is the last screening before diagnosis. SDCs were defined as cancer detected within three months after the index screening, while LICs were defined as cancer detected between C (length of the hypothetical screening interval) and the next screening. In this study, C was chosen to be 18 or 12 months. S_1_ is the next screening after the index screen, and S is the length of the current screening interval. (b) LICs diagnosed at T between C and S could be detected earlier by screening at the HS and (c) SDCs that were detected at index screening S_0_ could have been detected earlier at the HS. S_-1_ is the previous screening.

Late interval cancer cases defined by C<T<S might have benefitted from a shorter screening interval, where T is the individual time from index screen to diagnosis, S is the length of the actual screening interval, and C is the length of the assumed shorter screening interval (Figure 2(a)). We also assumed that these cases had been offered a hypothetical screening (HS) at time C after the index screen (Figure 2(b)). Cases detected at the index screen, defined as 0≤T≤3 (months, continuous time), might also have benefitted from a shorter screening interval (Figure 2(a)). The delay between screening and diagnosis is due to time for the clinical work-up. For these cases, we assume that they were offered an HS at time S-C before the index screen (Figure 2(c)). To have had a chance to avoid death from breast cancer, it was also necessary that the women in these groups should have participated in the HS and that the cancer was possible to detect with mammography.

Lead time is the time between the preclinical diagnosis at a screening test, e.g. mammography, and the hypothetical diagnosis of the same cancer detected clinically in the absence of screening. The late-interval cancer cases would have a lead time in the interval $\left(0,\;S-C\right)$ if the cancer was detected at the HS, otherwise it would be unchanged as 0. The screen-detected cases would get their lead time extended by S-C, if the cancer was detected at the HS; otherwise, it would be unchanged. Thus, for cases detected at the HS, the extended lead time might be larger for the screen-detected cancers.

Sojourn time is the preclinical period when the cancer is detectable with mammography but has not become clinically detectable, i.e. the maximum lead time. We calculated the expected lead time for a given sojourn time and for a constant screening interval length by simulation. Each simulated woman was given a fixed interval with start point when the tumor became detectable by mammography and end point when the cancer became symptomatic and would have been clinically detected, i.e. the length of each interval was the sojourn time. The end point of the interval was simulated between two consecutive screenings using a uniform distribution (0, L), where L is the length of the screening interval. The time points for individual screening invitations could then be calculated. We also allowed for a specific sensitivity (the probability to detect a detectable tumor at screening) by simulating the number of times a tumor would be missed at the screening occasions for each woman from a geometric distribution. The actual lead time could then be calculated for every simulated woman.

The mortality reduction achieved by using a shorter interval on the late interval and screen-detected cancer cases who died of breast cancer is unknown, but such a reduction will probably strongly depend on the length of the increased lead time. However, by making assumptions about the mortality reduction among the deceased cases who had potential to benefit from screening at HS, 1−$R_{C}$, the gain from shortening the screening interval from S to C given$\;R_{C}$ can be calculated. Let X be the total number of breast cancer deaths among participants in the study group and $P_{C}$ be the proportion of these with C<T<S or 0≤T≤ three months, i.e. the two groups with potential to benefit from changing the interval from S to C. The number of such cases is$\;P_{C}X$. $P_{C}$ was based on the 511 breast cancer deaths with complete data (Table 1), while X was equal to the 547 participants.

**Table 1.** Number and percent of women with complete data who participated in the index screening and who died from breast cancer by time in months (continuous time) from screening to diagnosis.

Time (T) from screening in the index round to diagnosis (months)	No. of breast cancer deaths (participated)	Percent (%)
0–3, index round first screening	81	15.9
0–3, index round subsequent screening	130	25.4
3^+^–12	118	23.1
12^+^–18	100	19.6
18^+^–24	58	11.3
24^+^–26	9	1.8
26^+^	15	2.9
Total	511	100

The effectiveness for participants in the screening program already estimated as the rate ratio $R_{T}=$ 0.71^7^ can be written as$\;R_{T}=X/E$, where E is the expected number of breast cancer deaths in the study group without screening. The new screening effectiveness using the interval length C is${\;R}_{TC}=\left(X-P_{C}X\left(1-R_{C}\right)\right)/E=\left(1-P_{C}\left(1-R_{C}\right)\right)R_{T}\;$.

The relative effectiveness of the screening program $R_{TC}/R_{T}=1-P_{C}\left(1-R_{C}\right)$ is thus independent of${\;R}_{T}$. For the definition of interval cancer cases, we assumed *S* to be 26 months even if *S* varied between counties.

The expected number of breast cancer deaths avoided is ${E_{da}=P}_{C}X\left(1-R_{C}\right)$ so ${\;R}_{TC}=\frac{\left(X-E_{da}\right)}{X}R_{T}$ and $R_{TC}/R_{T}=1-\frac{E_{da}}{X}$. Thus, if $E_{da}$ is calculated separately for screen-detected and interval cancer, the expected numbers can be added to calculate the combined effectiveness ($R_{TC}$) and relative effectiveness ($R_{TC}/R_{T}$).

We estimated the effect of shortening the screening interval from the 21-month average in the SCRY study to 18 or 12 months, assuming a breast cancer mortality reduction (1 − R_c_) from 5% up to 30% for the interval cancer cases and screen-detected cancer cases who died from breast cancer.

## Results

Out of the 511 women participating in the index round and who died from breast cancer, 58 cases diagnosed clinically at 18^+^–24 months (continuous time) after the index screening and 9 cases diagnosed after 24^+^–26 months were defined as late interval cancers. These 67 cancer cases would have had a chance to be detected at screening if an 18-month interval had been used. With the assumption of a 12-month interval, an additional 100 interval cancer cases diagnosed at 12^+^–18 months after the index screen would have had a chance to be screen detected. Furthermore, 130 screen-detected cancer cases whose index screening was a subsequent screening could also have been detected earlier if a shorter interval had been adopted (Table 1). Thus, 38.5% of the breast cancer deaths (13.1% interval cancer cases and 25.4% screen-detected cases) had a potential chance to be detected earlier using an 18-month interval. With a 12-month interval, the corresponding number was 58.1% (32.7% interval cancer cases and 25.4% screen-detected cases).

Assuming an 18-month screening interval and only considering interval cancers, the relative effectiveness ($R_{TC}/R_{T}$) varied from 0.961 to 0.993 when $R_{C}$ varied from 0.70 to 0.95 (30% to 5% mortality reduction), i.e. there was an additional reduction in the breast cancer mortality of 0.7%–3.9%, and the total estimated effectiveness, $R_{TC}$, was in the interval 0.682–0.705 (Table 2). Considering only screen-detected cancers at subsequent screenings, the relative effectiveness varied from 0.924 to 0.987 and the total effectiveness of the screening program, $R_{TC}$, was in the interval 0.656–0.701. Considering both interval cancers and screen-detected cancers at subsequent screening, the corresponding figures were 0.885–0.981 and 0.628–0.696, respectively (Table 2).

**Table 2.** Mortality results using a hypothetical screening interval of 18 or 12 months compared to the actual results of the SCRY study (with screening interval 21 months on average).

Assumed mortalityreduction (MR) (%)	Expected no. of breast cancer deaths avoided (_Eda_)	Relative effectiveness compared to the SCRY study (ReRR)	Effectiveness (R_TC_)
Hypothetical length of screening interval: 18 months			
Interval cancer *only*			
30	21.5	0.961	0.682
25	17.9	0.967	0.687
20	14.3	0.974	0.691
15	10.7	0.980	0.696
10	7.2	0.987	0.701
5	3.6	0.993	0.705
Subsequent screen-detected cancer *only*^a^			
30	41.7	0.924	0.656
25	34.7	0.937	0.665
20	27.8	0.949	0.674
15	20.8	0.962	0.683
10	13.9	0.975	0.692
5	6.9	0.987	0.701
Interval cancer *and* subsequent screen-detected cancer			
30	63.2	0.885	0.628
25	52.6	0.904	0.642
20	42.1	0.923	0.655
15	31.6	0.942	0.669
10	21.1	0.962	0.683
5	10.5	0.981	0.696
Hypothetical length of screening interval: 12 months			
Interval cancer *only*			
30	53.7	0.902	0.640
25	44.7	0.918	0.652
20	35.8	0.935	0.664
15	26.8	0.951	0.675
10	17.9	0.967	0.687
5	8.9	0.984	0.698
Interval cancer and subsequent screen-detected cancer			
30	95.3	0.826	0.586
25	79.5	0.855	0.607
20	63.6	0.884	0.627
15	47.6	0.913	0.648
10	31.8	0.942	0.669
5	15.9	0.971	0.689

a The effect on subsequent screen-detected cancer *only* using a 12-month screening interval is the *same* as using the 18-month screening interval.

R_C_: assumption of relative mortality among cases who could benefit from using screening interval length C compared to S; R_TC_: Effectiveness of using screening interval length C; SCRY: SCReening of Young women.

MR = (1 − R_C_) × 100.

R_T_ = 0.71 (effectiveness estimated in the SCRY study).

ReRR = R_TC_/R_T._

With a 12-month screening interval, the effect on the screen-detected cancers for a given $R_{C}$ was the same, but considering interval cancers, the relative effectiveness varied from 0.902 to 0.984 when $R_{C}$ varied from 0.70 to 0.95. Considering both interval and screen-detected cancers at subsequent screenings, the relative effectiveness varied from 0.826 to 0.971 (Table 2).

**Figure 3.**
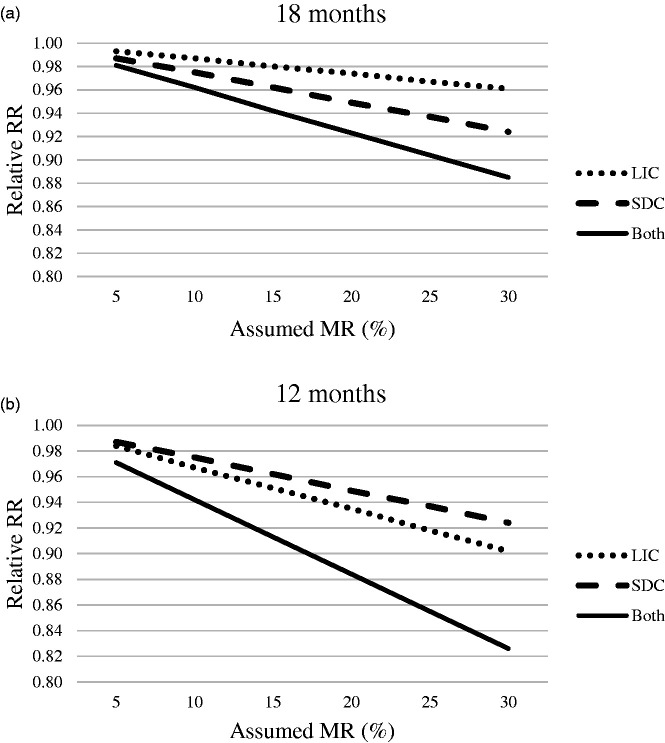
This plot illustrates the relative effectiveness (relative RR) of hypothetically using a shorter screening interval given an assumed mortality reduction (MR) on those late interval cancer cases (LIC), subsequent screen-detected cancer cases (SDC), and the combination of the two who died from breast cancer in the actual screening program. The dashed line indicates subsequent screen-detected cancers, the dotted line indicates late interval cancers, and the solid line indicates both. (a) Hypothetical screening interval of 18 months; (b) Hypothetical screening interval of 12 months.

The potential effect of changing to a shorter screening interval of 18 or 12 months was increased when $R_{C}$ varied from 95% to 70%. With an 18-month screening interval, the effect was larger for screen-detected cancers than for late interval cancers, and the situation was the opposite with a 12-month screening interval (Figure 3(a) and (b)).

It is possible to calculate the effectiveness measures using different mortality reductions (${1-R}_{C}$) for screen-detected and interval cancer cases. As an example, assume an 18-month screening interval and mortality reduction at 20% and 10% for interval cancer and subsequent screen-detected cancer, respectively. In Table 2, we find the expected number of breast cancer deaths avoided (Eda) to be 14.3 and 13.9, respectively, giving a total of 28.2. The combined effectiveness is then ${\;R}_{TC}=\frac{\left(547\;-\;28.2\right)}{547}0.71\;=\;0.673$ and the combined relative effectiveness $R_{TC}/R_{T}=1-\frac{28.2}{547}=0.948$

For a screen-detected case with a sojourn time of 2.4 years (which has been estimated as the average for the age group 40–49 years),^16^ a 21-month screening interval, and 70% sensitivity, the expected lead time calculated by simulation was 16.9 months. Thus, for the screen-detected deceased cases considered above, it would be possible to increase the lead time by 3 months to 19.9 months (18%) for an 18-month interval and by 9 months to 25.9 months (53%) for a 12-month interval. For comparison, lead time for the late interval cancer cases could be increased from 0 to 0–3 months for an 18-month interval and to 0–9 months for a 12-month interval depending on the distribution of the time to diagnosis.

## Discussion

Data from the SCRY study were used to calculate the hypothetical gain of a shorter screening interval for women aged 40–49 years who had participated in screening and had died from breast cancer. We utilized the effectiveness of screening already estimated in SCRY and calculated the proportion of breast cancer deaths that would have been avoided with a shorter screening interval. Within a range of different assumed mortality reductions for these deceased cases, the number of potentially avoided deaths and the effectiveness relative to SCRY was calculated. Assuming a 15% mortality reduction if an 18-month interval had been used, this would have increased the effectiveness by 6% relative to a 21-month interval, and an additional 32 breast cancer deaths could have been avoided (11 interval cancers and 21 screen-detected cases). Using a 12-month interval, the corresponding figures were a 9% increase in effectiveness and 48 avoided breast cancer deaths (27 interval cancers and 21 screen-detected cases) (Table 2).

Data and results from the Swedish nationwide SCRY study were used, and this study was larger than all randomized studies combined for the age group 40–49 years.^7^ Many studies on screening interval length have focused on differences in tumor characteristics and detection mode, but the present study focused on the primary aim of screening, i.e. a reduction in breast cancer mortality. The screening intervals in SCRY varied between counties from 18 to 24 months, but our results are based on the average of 21 months.

Among the 15 cases with breast cancer diagnosis >26 months after the index screening, 8 were diagnosed within 26^+^–29 months. A probable explanation for this is abnormally long screening intervals for short periods of time due to, for example, manpower shortages in the program or individual migration within areas that offer mobile screening.

Although we have identified cases that could potentially have benefitted from screening at shorter intervals, we do not know what would have been the effect of earlier screening of these cases. The main factor having an impact on this is the increased lead time, which in turn depends on participation and sensitivity at the HS and on the sojourn time. A shorter screening interval may for some cases lead to a longer lead time, while for others it would be unchanged. However, for some screen-detected cases, an earlier screening may even result in a shorter lead time if the cancer had not progressed enough at that time point and therefore was not detected. In this study, the results were not based on individual increase of lead time but on the assumed mortality reduction due to the hypothetical screening for the whole group.

We believe that the increase in lead time is relatively small compared to the average lead time for screen-detected cancers in SCRY, and we have therefore presented the results for a varying mortality reduction due to the hypothetical screening with the effectiveness in SCRY ($R_{T}$) set as the maximum.

In the calculations of the combined effect of both interval cancer and screen-detected cancer in Table 2, we assumed a similar reduction in mortality in the HS for interval cancer and screen-detected cancer.

In the current study, we only considered cases who died from breast cancer. This can imply a selection bias towards cases with shorter lead times. However, the simulated lead time calculations were made for average screening-detected cases.

## Conclusion

The potential effect of shortening the screening interval in women aged 40–49 years to 18 or 12 months might further reduce breast cancer mortality. With a moderate change in the interval from 21 to 18 months, most of the extra effect was due to lives saved among screen-detected cancer, but with a 12-month interval, the effect on interval cancer was stronger.
